# Anti‐Seizure Medications Alter Functional and Effective Connectivity as Measured With Intracranial Electroencephalography

**DOI:** 10.1002/brb3.71507

**Published:** 2026-05-27

**Authors:** Serdar Akkol, Helen E. Brinyark, Rebekah Chatfield, Aparna Vaddiparti, Rachel June Smith, Benjamin C. Cox

**Affiliations:** ^1^ Department of Neurology University of Alabama at Birmingham Birmingham Alabama USA; ^2^ Department of Biomedical Engineering University of Alabama at Birmingham Birmingham Alabama USA; ^3^ Department of Electrical and Computer Engineering University of Alabama at Birmingham Birmingham Alabama USA; ^4^ Birmingham VA Medical Center Neurology Service Birmingham Alabama USA

**Keywords:** broadband high frequency activity, graph theoretical measures, medication side effect, non‐involved zone, seizure onset zone

## Abstract

**Background:**

Anti‐seizure medications (ASMs) control seizures through distinct neuronal mechanisms. While suppressing seizures, putative spill‐over effects can lead to multiple cognitive side effects, some of which can be explained by the modulation of brain connectivity. This modulation can be studied with functional and effective connectivity. While functional connectivity provides information on statistical dependencies between two brain regions, effective connectivity as measured with cortico‐cortical evoked potentials (CCEPs) through single pulse electrical stimulation (SPES) can elucidate the underlying mechanisms of the effective connectivity with high temporal and spatial resolution. CCEPs are often performed while patients are on ASMs; however, the effect of ASMs on CCEPs is largely unknown given limited opportunity to experimentally study this in the human brain. We hypothesized that ASMs would alter the connectivity within the seizure network more often than in healthy brain areas.

**Objective:**

We aimed to understand the effects of ASMs on functional and effective connectivity.

**Methods:**

We recruited seven patients undergoing invasive monitoring with depth electrodes for medically refractory epilepsy, five of whom underwent SPES with 5 mA, 150 µsec per phase square wave pulses at 1 Hz frequency while the patients were on ASMs as part of ongoing research projects (ASM‐ON). Since patients did not have their typical seizures, a second SPES session was performed by the clinical team while off ASMs to reduce their seizure threshold (ASM‐OFF). We recorded a total of 565 bipolar channels and electrically stimulated a total of 17 seizure onset zone (SOZ), 6 early propagation zone (EPZ), 36 irritative zone (IZ), and 64 non‐involved zone (NIZ) electrode pairs across five patients. We compared the amplitude and latency of early and late voltage deflections (N1, N2) and root mean square values of CCEPs between two sessions. In a partly overlapping cohort of five patients, we also recorded 1‐h long rest sessions (ASM‐ON and ASM‐OFF) to show the changes in functional connectivity and graph theoretical measures obtained from the slow fluctuations in broadband high frequency activity.

**Results:**

ASMs preferentially modulated excitability within the epileptic network, with the highest rates of significant CCEP amplitude changes observed for seizure network (SOZ→SOZ: 15.9% N1, 15.5% N2; EPZ→EPZ: 15.6% N1, 11.1% N2; IZ→IZ: 11.6% N1, 12.4% N2) compared to NIZ→NIZ (5.7% N1, 7.4% N2), and with amplitude effects consistently exceeding latency effects across all tissue class combinations (*p* < 0.05, group significance at *q* < 0.05). We also found that the RMS based connectivity and functional connectivity were altered more often outside of the seizure network.

**Conclusions:**

Overall, we found that ASMs altered the effective connectivity within the seizure network more than within non‐involved regions, whereas functional connectivity was altered more often within non‐involved regions. This study is the first study revealing with high spatiotemporal resolution that ASMs can alter brain effective and functional connectivity in multiple different ways.

## Graphical abstract

ASMs affected the effective connectivity within the seizure network more than in the healthy network. Alterations in functional connectivity were found within the healthy network.

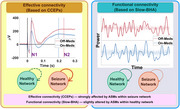

## 1 Introduction

Epilepsy affects more than 45 million people worldwide (Beghi et al. 2019) and is one of the top five neurological diseases to cause significant global burden as measured by disability‐adjusted life‐years (Feigin et al. 2017). Virtually all patients require maintenance anti‐seizure medications (ASMs) and at least 20% are on polytherapy with many experiencing adverse effects (Chen et al. 2018). Although ASMs utilize multiple mechanisms to suppress seizures (Sills and Rogawski 2020), they lead to common adverse effects like drowsiness, fatigue, insomnia, dizziness, imbalance, ataxia, and paresthesias, along with other neuropsychiatric side effects including irritability, aggressive behaviors, and depression (Perucca and Gilliam 2012). Several ASMs can also work synergistically to control seizures while potentially increasing the risks of side effects (Perucca and Kwan 2005; Czuczwar et al. 2009; Canevini et al. 2010). However, little is known about the underlying mechanisms and their relation to the alterations in brain networks.

The brain can be seen as functionally and anatomically connected hierarchical nodes at the micro (e.g., neuronal) and macro (e.g., anatomical brain regions) levels (Stam and Reijneveld 2007; Friston 2011). In this context, functional connectivity is defined as the statistical dependency between temporally similar neurophysiological activities of separate brain regions (Biswal et al. 1995). The advancement of stereo‐EEG (sEEG) has opened the window to study the complex neuronal networks that give rise to seizures. Graph theory provides a tool to model the functional connectivity of these networks as a series of nodes and edges, which can demonstrate changes in these networks under different conditions (Boccaletti et al. 2006; Panzica et al. 2013). Functional connectivity and graph theoretical measures provide substantial understanding of how the brain networks work together and show the underlying mechanisms of how the brain nodes influence one another. While there are multiple statistical and mathematical tools (e.g., Granger causality) to study the effects of brain regions on each other, the biological or neurophysiological generalizability of these methods are limited due to the dependency only on the statistical correlations between activities (Mohanty et al. 2020; Cao et al. 2022). To study effective connectivity, electrical, magnetic, or optogenetic stimulation have been used more often with new neurotechnologies (Ilmoniemi et al. 1997; Matsumoto et al. 2004; Bauer et al. 2018).

Single pulse electrical stimulation (SPES) elicits a localized response at the stimulation site as well as an evoked potential in other cortical regions, termed cortico‐cortical evoked potentials (CCEPs) (Matsumoto et al. 2004). CCEPs have been studied to localize the seizure onset zone (SOZ) or to uncover directed causal network interactions and effective connectivity between brain regions (Keller et al. 2014b; Matsumoto et al. 2017). Each CCEP contains two main peaks. The first is the early negative response within 10–30 ms called the N1 peak and a later response within 80–250 ms called the N2 peak (Matsumoto et al. 2007). While the N1 is thought to be the result of the early excitation of pyramidal cells, the N2 is the average of long‐lasting net inhibition (Keller et al. 2014b). SPES sessions are typically conducted when patients are on ASMs, since SPES by delivering current to the brain can also trigger a seizure (Trébuchon and Chauvel 2016). On rare occasions, such stimulation can be applied when the patient is off of ASMs if the patient has not had typical seizures despite prolonged monitoring (Trébuchon and Chauvel 2016; Cuello Oderiz et al. 2019; Arya et al. 2025). To the best of our knowledge, there have been no prior studies investigating the effects of ASMs on effective connectivity.

Studies using functional magnetic resonance imaging (fMRI) and cortical thickness MRI have shown the effects of ASMs on cognitive networks (Wandschneider and Koepp 2016; Sammarra et al. 2024). Several studies found that ASMs attenuates activation and/or deactivation in the anatomical structures critical for the cognitive task including language or memory (Jokeit et al. 2001; Szaflarski and Allendorfer 2012; Wandschneider et al. 2014; Wandschneider et al. 2017). Additionally, certain ASMs have also been studied in their effects on functional connectivity (Haneef et al. 2015; Pedersen et al. 2024). However, most of these studies were correlational and did not assess the impact of ASMs within individuals. There are very few opportunities to study the effects of ASMs on functional connectivity in humans, since it would be unethical to stop ASMs for research studies. However, a subset of epilepsy patients undergo video‐ or intracranial electroencephalography (vEEG or iEEG) monitoring during which the ASMs are stopped for clinical reasons to record their habitual seizures and this period provides an opportunity to study the brain network level alterations (Spencer et al. 2008; Zaveri et al. 2010; Goncharova et al. 2016; Paulo et al. 2022).

Here, we used a unique opportunity in seven patients who underwent iEEG when their ASMs were tapered and discontinued as part of the clinical monitoring. We recorded CCEPs while five of the seven patients were on ASMs for ongoing research studies (ASM‐ON). Due to lack of their typical seizures, a second SPES session was performed by the clinical team, while they were off ASMs (ASM‐OFF). We studied the changes in amplitude and latency of N1 and N2 responses and root mean square (RMS). We also recorded 1 h resting state recordings in five patients (three of CCEP cohort and two additional) immediately before the SPES sessions and analyzed the functional connectivity and the graph theoretical measures of the slow fluctuations of broadband high frequency activity (BHA). We hypothesized that the effects of ASMs are more specific on the epileptic networks than the healthy networks.

## 2 Methods

### 2.1 Patients

We recorded sEEG on seven patients who were admitted to the Epilepsy Monitoring Unit (EMU) at the University of Alabama at Birmingham (Table 1). All patients provided written consent to participate in this study. A total of two SPES sessions were conducted: one clinical‐focused and one research‐focused, both by the respective teams. The first SPES session was conducted by the research team while patients were on their ASMs (ASM‐ON) using the experimental protocol that was reviewed and approved by the University of Alabama at Birmingham Institutional Review Board (IRB‐300009963). All procedures involving human participants were conducted in accordance with the ethical standards of the institutional research committee and the Declaration of Helsinki. In five of the seven cases, the clinical team decided to conduct a second SPES session once patients were off ASMs to potentially reduce their seizure threshold and/or induce seizures (ASM‐OFF). This session was conducted with patients’ clinical consent and decision. Stimulation contacts were chosen by the clinical team to assess epileptogenicity and seizure‐inducing potential. This led to variability in the number of stimulated contacts in both sessions. The ASM‐OFF session was conducted after at least five half‐lives of the ASM with the longest half‐life as 94%–97% of a drug is eliminated from the body at this time point except P6 (detailed in Section 3.1). The epileptic network was identified by the clinical team with the SOZ as the primary origin of seizures and the early propagation zone (EPZ) as the location where the seizures were initially spread before generalization. The irritative zone (IZ) was defined as the locations where the spontaneous interictal spikes and/or electrical stimulation induced after‐discharges were seen, but excluding SOZ and EPZ contacts. All other locations were identified as non‐involved zones (NIZ).

**TABLE 1:** Patient demographics and medications. Patients (P1–P7) had a variable history of epilepsy and seizure onset zone.

Patient	Age	Gender	Epilepsy age of onset	Days between ASM‐ON and ASM‐OFF	ASMs during ASM‐ON session	Seizure onset zone	Analyses
P1	37	F	35	19	LEV 2500 mg BID, VPA 500 mg BID, OXC 1200 mg BID	Right basal temporal cortex	CCEP only
P2	64	F	18	14	LEV 1500 mg BID, LTG 150 mg BID	Left posterior basilar temporal cortex and temporoparietal junction	CCEP and FC
P3	36	F	25	8	LEV 1250 mg AM and 750 mg PM, LTG 150 mg BID, ESL 400 mg daily	Left basal temporal region	CCEP only
P4	32	M	19	16	LEV 1500 mg AM and 500 mg PM, LTG 100 mg AM and 50 mg PM, LCM 100 mg AM and 50 mg PM	Right posterior hippocampus	CCEP and FC
P5	23	F	18	2	CNB 350 mg nightly, LTG 350 mg nightly, OXC 450 mg nightly	Left posterior insula and temporal operculum	FC only
P6	49	M	15	2	CNB 100 mg once, ZNS 100 mg BID, CLB 5 mg nightly	Left superior temporal gyrus, inferior parietal lobule	FC only
P7	47	F	4	4	BRV 200 mg BID, OXC 900 mg BID, LCM 200 mg BID	Right hippocampus, amygdala, superior and middle frontal gyrus	CCEP and FC

Abbreviations: ASM, anti‐seizure medication; BID, bid in die (twice daily); BRV, brivaracetam; CNB, cenobamate; ESL, eslicarbazepine; FC, functional connectivity; LEV, levetiracetam; LTG, lamotrigine; OXC, oxcarbazepine; VPA, valproic acid.

### 2.2 Electrodes and Localization

All patients were implanted with depth electrodes (PMT Corporation, Chanhassen, Minnesota, USA for P1 and P2; AdTech Medical, Oak Creek, Wisconsin, USA for P3 to P7). The recordings were acquired with an XLTEK Quantum Amplifier (Natus Medical Inc., San Carlos, California, USA), digitized at 2048 Hz. A subdural or subdermal contact was used as a reference contact. sEEG electrodes were co‐registered to the patient's presurgical MRI from their postimplantation CT scan using Curry 9 software (Neuroscan Compumedics, Charlotte, North Carolina, USA).

### 2.3 SPES

Electrical stimulations were given through a pair of adjacent electrodes to deliver current using Nicolet Macrostimulator (Natus Medical Inc., Middleton, Wisconsin, USA). Each stimulation was 5 mA, 150 µsec per phase biphasic, amplitude‐balanced, square‐wave pulses at 1 Hz. Each stimulation train consisted of 30–120 pulses. All pulses were included in the analyses.

### 2.4 sEEG Data Acquisition and Preprocessing

Recordings where the patients had clinical seizures were excluded from the analysis. The sEEG data were preprocessed using Fieldtrip (Oostenveld et al. 2011) and custom MATLAB scripts (R2023a, MathWorks, Natick, Massachusetts, USA). First, notch filtering was performed to remove line noise and its harmonics (with zero‐phase, third order, Butterworth filter with band‐stops between 59–61, 119–121, and 179–181 Hz). For CCEP analyses, we chose bipolar referencing by subtracting the monopolar signals of two adjacent contacts of the same electrode. Noisy/corrupted channels were manually excluded from further analysis steps. Stimulation artifact was then identified either using the stimulation contact or the closest contact on the same electrode wire to get the most accurate stimulation onset timing. Given that electrical stimulation may induce changes in impedance, we normalized each period by subtracting the average voltage values of baseline (between −215 ms and −15 ms time‐locked to each electrical stimulation pulse) from the poststimulation window.

### 2.5 CCEP Parameters: N1, N2, and RMS

Within their respective windows, we found the local peak absolute values of N1 and N2 for each individual CCEP (in single trials) at each distant location using the “findpeak” function of MATLAB. N1 responses were between 10 and 30 ms while N2 responses were between 85 and 250 ms (Matsumoto et al. 2004, 2007; Enatsu et al. 2013). We did not assign any values if there was no peak found. We also used RMS as part of the effective connectivity analyses. RMS has been a widely used, sensitive, and established tool to calculate the effective connectivity between pairs of sites (Enatsu et al. 2013; Prime et al. 2020). It was calculated as the square root of squares of average CCEP voltage values divided by the length of time from 10 to 300 ms (1 ms slide) for each averaged response. RMS is also divided into two portions, an early response between 10 and 50 ms and a later response between 50 and 300 ms.

### 2.6 Calculating Functional Connectivity and Graph Theoretical Measures

To show how functional connectivity changes across two sessions, we obtained 1‐h resting state iEEG data in five patients (P2, P4, P5, P6, and P7) (Table 1) immediately prior to the CCEP session. The other two patients did not have resting state data in either of the times (ASM‐ON or ASM‐OFF). Here, our goal was (1) to show the functional connectivity within different networks (epileptic vs. healthy) and (2) to describe the changes of connectivity from ASM‐ON to ASM‐OFF and which networks are most affected by the ASM. To obtain functional connectivity through iEEG data, we correlated the low frequency (0.1–1 Hz) filtered signal of BHA between each contact. Low‐frequency filtered BHA is temporally correlated with population spiking activity and with fMRI‐based network topography and this method has been used previously both for healthy and abnormal epileptogenic tissues (Biswal et al. 1995; Nir et al. 2008; Manning et al. 2009; Keller et al. 2013; Kucyi et al. 2018; Leszczyński et al. 2020; Akkol et al. 2021).

First, we calculated the common average re‐referencing excluding channels that were in SOZ or EPZ or were manually selected for being noisy or corrupt. Time‐frequency decomposition with frequencies log‐spaced between 70 and 200 Hz (14 values) using the Morlet transform at 10 ms steps with seven cycles. For power spectral analysis, the absolute value of the Morlet output (power) was rescaled with log normalization at each frequency and then averaged across all frequencies to obtain BHA. Subsequently, a bandpass filter (zero‐phase, Butterworth, third order) was applied to BHA retaining the frequencies between 0.1 and 1 Hz. This low frequency signal of BHA was used to calculate Pearson's pairwise‐correlation between each contact to obtain the functional connectivity estimate.

We then calculated graph theoretical network measures to characterize the differences in the network topology between ASM‐ON and ASM‐OFF. After constructing the absolute of the functional connectivity matrices as calculated above, weighted measures were employed to preserve the continuous nature of the connectivity data. To generate adjacency matrices, the diagonal is set to zero and a threshold was set at the 40th percentile of combined connectivity values from both sessions. We used Brain Connectivity Toolbox to calculate several network metrics to capture various aspects of brain network organization: degree, strength, clustering coefficient, path length, modularity, global and local efficiency, and small‐worldness (Rubinov and Sporns 2010). Degree is the number of direct connections a node has toward other nodes. Strength is the sum of all connection weights of a node (i.e., the total intensity of its connections). Clustering coefficient is a measure of how much a node's neighbors are connected to each other, reflecting the local segregation or cohesiveness. Path length is the average number of steps along the shortest paths between all possible pairs of nodes, quantifying network integration, while modularity measures how well a network can be divided into distinct modules with dense internal connections and sparse external connections. Global efficiency is the average inverse shortest path length between all node pairs, representing the capacity for efficient information transfer across the network. On the other hand, local efficiency reflects the average efficiency of information transfer within the local neighborhoods of individual nodes. Small‐worldness is characterized by high clustering and short path lengths, indicating an optimal balance between segregation and integration for efficient information processing. Statistical comparisons for graph theoretical measures between ASM‐ON and ASM‐OFF were conducted using Wilcoxon signed‐rank tests with *p* < 0.05 when appropriate.

### 2.7 Statistical Analyses on Effective and Functional Connectivity

To identify the significant changes in amplitude and latency of CCEP N1 and N2 between ASM‐OFF and ASM‐ON states, a permutation‐based hypothesis test was used at the level of individual pairs. For each stimulated channel—recording channel pair and each measure (combination of N1/N2 and amplitude/latency), the observed difference in the means (ASM‐OFF minus ASM‐ON) was computed across single trials. A null distribution was constructed by randomly permuting trial labels between conditions (ASM‐ON vs. ASM‐OFF) 5000 times and recomputing the mean difference at each iteration, yielding an empirical two‐sided *p*‐value. To control for the large number of simultaneously tested pairs, *p*‐values were corrected for multiple comparisons separately for each measure using the Benjamini–Hochberg false discovery rate procedure (FDR) (*q* < 0.05). Significant pairs were further classified by direction of effect (increased or decreased relative to ASM‐ON) and summarized according to the epileptogenic profile of the stimulated and recording electrodes (SOZ, EPZ, IZ, NIZ). Thus, stimulation–recording channel pairs that have percentages higher than 5% were deemed to be significantly different between conditions. We acknowledge that due to the small number of patients and channels, we were not able to fully control for the patient ID in this analysis.

We compared the latencies and amplitudes of N1 and N2 peaks and the RMS values obtained from ASM‐OFF and ASM‐ON sessions by fitting a linear regression for each stimulation and recording channel pair to compare the effect of ASM on the functional connectivity. The model specification was:

$$RMS∼State_{ASM}+PatientID$$

where $State_{ASM}$ is a binary categorical variable (OFF = 0, ON = 1) and PatientID accounts for subject‐level random variation as a main effect. This approach allows estimation of the population‐level effect of medication state while controlling for between‐subject variability. Multiple comparisons were controlled using family‐wise error correction (Bonferroni method, *α* = 0.05/48), accounting for 48 total statistical tests (3 metrics × 16 stim‐rec pairs). Effects were declared significant if *p* < 0.00104.

To statistically compare the functional connectivity between two rest sessions (ASM‐ON and ASM‐OFF), we employed a nonparametric permutation testing. For each electrode pair, we calculated the observed absolute difference between the correlation coefficients in the ASM‐ON ($r_{ASM-ON}\left(i,j\right)$) and ASM‐OFF ($r_{ASM-OFF}\left(i,j\right)$) conditions: $T_{observed}\;\left(i,j\right)=\left|r_{ASM-ON}\left(i,j\right)-r_{ASM-OFF}\left(i,j\right)\right|$. To construct the null distribution, we performed 10,000 permutations. In each permutation, the condition labels (ASM‐ON or ASM‐OFF) were randomly shuffled across the original time series data and correlation matrices were recalculated using the permuted data followed by calculation of test statistic $T_{permuted}\left(i,j\right)$ using the same formula as for the observed data. For each electrode pair, an empirical *p*‐value was calculated as the proportion of permutations in which the permuted test statistic exceeded or equaled the observed test statistic:

$$\begin{array}{ccc} p\;\left(i,j\right)= & & \left(1+\sum_{k=1}^{n_{perm}}I\left(T_{permuted,k}\left(i,j\right)\;\geq T_{observed}\left(i,j\right)\right)\right)\\ & & /\left(1+n_{perm}\right) \end{array}$$

where I(·) is the indicator function that equals 1 when the condition is met and 0 otherwise, and $n_{perm}=10,000$ is the number of permutations. To control the family‐wise error rate across the multiple electrode pairs, we recorded the maximum test statistic across all electrode pairs and the critical threshold was set at the 99th percentile of this maximum statistic distribution, thus only electrode pairs with observed test statistics exceeding this threshold were considered statistically significant (*p* < 0.01, Bonferroni corrected). In addition to channel‐level analyses for functional connectivity, we also compared the average functional connectivity between sessions. Here, we used Wilcoxon signed‐rank tests with alpha value of 0.05.

## 3 Results

### 3.1 Patients and Recordings

We recorded iEEG while seven patients were on and off ASMs. Patient demographics are summarized in Table 1 along with the contributions of CCEP and functional connectivity data from each patient.

The first patient (P1) was a 37‐year‐old female with epilepsy that started at 35 years old, who was implanted with sEEG electrodes for medically refractory focal epilepsy with the primary hypothesis of seizure onset in the right mesial temporal lobe. At the time of sEEG, her ASM regimen was levetiracetam (LEV) 2500 mg bid in die (BID [twice daily]), valproic acid (VPA) extended release (ER) 500 mg BID, and oxcarbazepine (OXC) 1200 mg BID. The ASM‐ON session was performed on the day after the sEEG implantation (Day 1) while she was on her home ASM regimen. The patient had been implanted for more than 2 weeks with only one electrographic (i.e., subclinical) seizure and so the ASM‐OFF session was performed on Day 20. This session was carried out by the clinical team 17 days after the patient's last ASM dose. With subsequent spontaneous seizures, the SOZ was determined to be in the right basal temporal, the EPZ in the right hippocampus and the IZ in the left hippocampus.

The second patient (P2) was a 64‐year‐old female with epilepsy that started at the age of 18. She was monitored in the EMU with the primary hypothesis of seizure onset in the left mesial temporal lobe. Prior to implantation, the patient was on LEV 1500 mg BID and lamotrigine (LTG) 150 mg BID. The initial CCEP session (ASM‐ON) was obtained the day after implantation (Day 1) while the patient was on home ASMs. Despite 2 weeks of monitoring, no spontaneous electroclinical seizures occurred. Thus, the second session (ASM‐OFF) was conducted on Day 14, 12 days after the patient's last ASM dose. The clinical team identified the SOZ in the left posterior basilar temporal cortex and temporo‐parietal junction, the EPZ in the right hippocampus, and the IZ in the left hippocampus.

The third patient (P3) was a 36‐year‐old female with epilepsy that started at the age of 35. She was implanted with the primary hypothesis of the left mesial or basal temporal region. Home ASM regimen was LEV 2250 mg in the morning and 1500 mg at night, LTG ER 600 mg nightly, and eslicarbazepine (ESL) 1200 mg daily. The ASM‐ON session was performed on the day after the sEEG implantation (Day 1), when the patient was given LEV 1250 mg in the morning and 750 mg at night, LTG 150 mg BID, and ESL 400 mg at night. The ASM‐OFF session was performed on Day 10, 6 days after the last dose of ASMs. Implantation revealed the SOZ in the left basal temporal region and IZ in the left neocortical and mesial temporal regions. She had a seizure induced during the SPES of a left anterior hippocampal electrode and this stimulation session was excluded from further analyses.

The fourth patient (P4) was a 32‐year‐old male with epilepsy that started at the age of 19. He was implanted with sEEG with the primary hypothesis of SOZ in right medial or anterior temporal, insular, or orbitofrontal regions. His home ASM regimen was LEV 2000 mg BID, LTG ER 250 mg BID, and lacosamide (LCM) 200 mg BID. The initial CCEP session was performed on the day after implantation (Day 1) when the patient received LEV 1500/500 mg, LTG 100/50 mg, and LCM 100/50 mg. On Day 17, the ASM‐OFF CCEP session was performed 14 days after the last ASMs were given (Day 3). During his monitoring, the patient did not have any habitual seizures, thus the clinical team epileptic networks were decided based on interictal findings and stimulation‐induced seizures. SOZ was determined to be the right posterior hippocampus and IZ was the right amygdala, right anterior hippocampus, and right temporal neocortex.

The fifth patient (P5) was a 23‐year‐old left‐handed woman with a history of anxiety and depression, and drug‐refractory focal onset epilepsy was implanted with the primary hypothesis of SOZ in left limbic structures. Her epilepsy began at age 18. Prior to admission, her ASM regimen consisted of cenobamate (CNB) 350 mg at bedtime, LTG 350 mg at bedtime, and OXC XR 900 mg at bedtime. On Day 1, during the ASM‐ON session, she was given CNB 350 mg at bedtime, LTG 350 mg at bedtime, and OXC 450 mg at bedtime. Her last medication (LTG 25 mg) was administered on Day 8 and the ASM‐OFF recording was on Day 10. During her evaluation, the SOZ was identified in the left posterior insula/temporal operculum, the EPZ was localized to the left mesial temporal and frontal cortex, and the IZ included the right insula.

The sixth patient (P6) was a 49‐year‐old left‐handed man with intellectual disability and drug and surgical refractory epilepsy was implanted with bilateral sEEG leads. Age at seizure onset was 15. The patient's home ASM regimen was CNB 200/25 mg, zonisamide (ZNS) 400 mg BID, and clobazam (CLB) 10 mg nightly. On the day of ASM‐ON session (Day 2), the patient received CNB 100 mg AM, ZNS 100 mg BID, and CLB 5 mg nightly. The ASM‐OFF was on Day 11 with the latest ASMs (CNB 50 mg and ZNS 100 mg) given on Day 9. SOZ was determined to be left superior temporal gyrus and inferior parietal lobule.

The seventh patient (P7) was a 47‐year‐old female with bipolar disorder, post‐traumatic stress disorder, anxiety, atrial septal defect, cerebral palsy due to premature birth with residual left‐sided spastic hemiparesis, and large right porencephalic cyst, and medically refractory focal epilepsy with VNS implanted with sEEG leads to localize SOZ around the cyst and mesial temporal structures. The patient was on brivaracetam (BRV) 200 mg BID, OXC 900 mg BID, and LCM 200 mg BID prior to admission. The ASM‐ON session was recorded on Day 2 while the patient was on her home ASMs. The last dose of ASM was OXC 150 mg on Day 6 and the ASM‐OFF session was recorded on Day 10. SOZ was deemed to be the right hippocampus, amygdala, and superior and middle frontal gyrus and EPZ was the right precuneus, posterior insula, and posterior cingulate. IZ was determined to be the right lateral temporal cortex and anterior insula.

For P1, we stimulated 1 EPZ and 4 IZ channels in both ASM‐ON and ASM‐OFF sessions. For P2, we stimulated 5 SOZ, 1 EPZ, 28 IZ, and 40 NIZ channels in both sessions. For P3, 2 SOZ and 4 NIZ were stimulated in both sessions. P4 had five stimulated electrodes in both sessions with three of them being SOZ, one being EPZ, and one being IZ. P7 had a total of 194 channels recorded and 7 SOZ, 4 EPZ, 4 IZ, and 21 NIZ channels were stimulated in both sessions. Across five patients, we recorded from 64 SOZ channels, 32 EPZ, 106 IZ, and 508 NIZ channels.

### 3.2 Effects of ASMs on CCEPs

**FIGURE 1:**
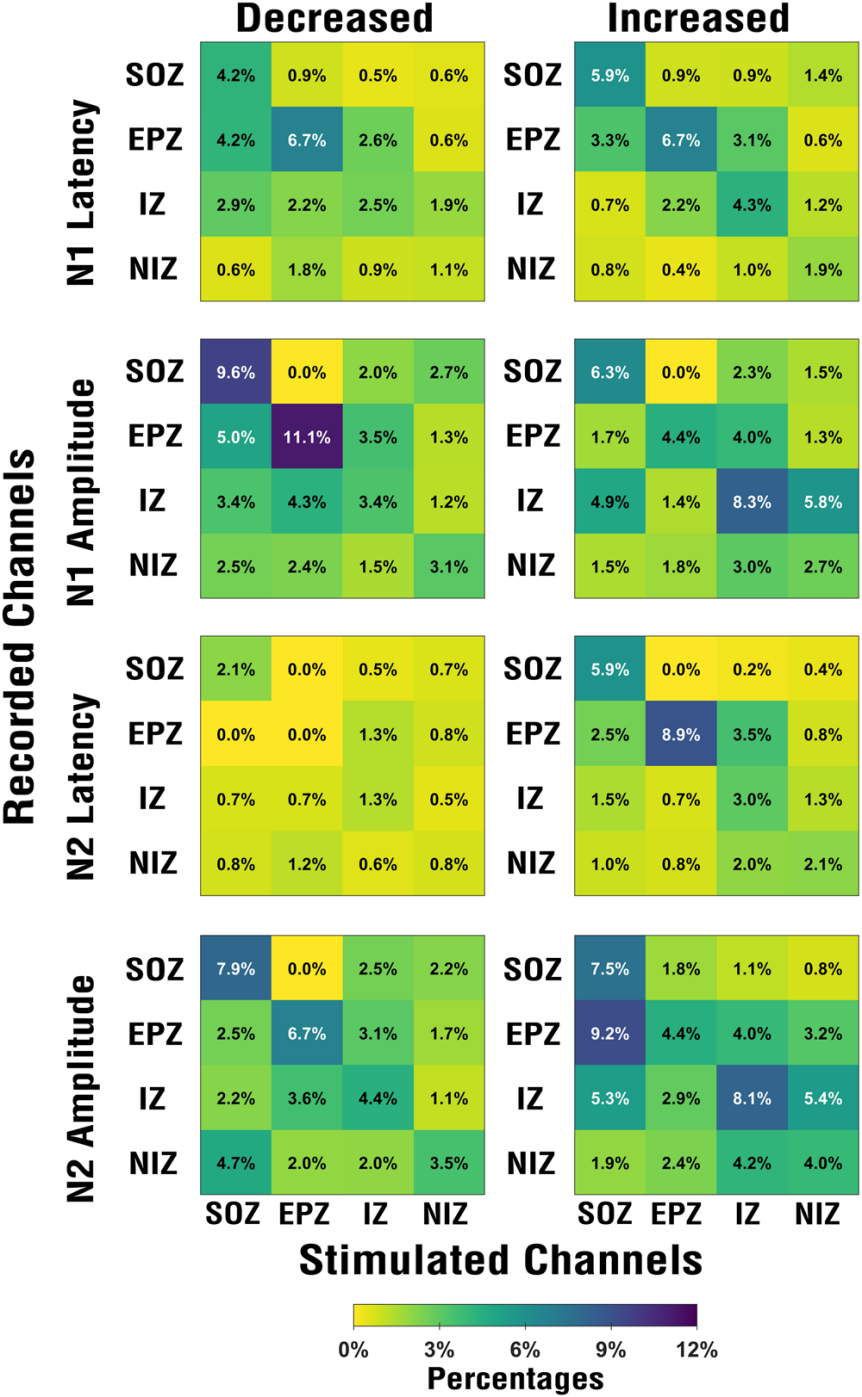
Changes in the latency and amplitude of N1 and N2 responses from comparing the ASM‐ON to ASM‐OFF session. Each box within each panel indicates the percentage of electrode pairs of the stimulation channels (in columns) and the recording channels (in rows) that showed significant change based on *t*‐test results. Four properties are displayed: N1 latency, N1 amplitude, N2 latency, and N2 amplitude. The left hand panels show the percentage of channel pairs with increase in the respective property, while the right hand panels show the percentage of channel pairs with decrease in the respective property. False discovery rate is 5%, thus values exceeding 5% are significantly increased/decreased than chance and are depicted with white font color.

After finding the N1 and N2 peaks in individual trials, we measured their latency and amplitude for each SPES. We then compared how the latency and amplitude changed from ASM‐ON to ASM‐OFF within each electrode with permutation testing and FDR correction for each comparison (*q* < 0.05). Given there were only a few number of channels stimulated in each patient, we combined the channels across patients and reported the percentage of recording channels that showed significant change (either decrease or increase in Figure 1). The resulting stimulation–recording channel pairs that contain more than 5% significantly altered pairs were deemed to be significantly different between ASM‐ON and ASM‐OFF. We found that the changes in latency or amplitude of N1 and N2 were mostly within the seizure network (SOZ, EPZ). For example, when SOZ were stimulated, N1 amplitude was decreased in 9.6% of SOZ recording channels and increased in 6.3% of SOZ recording channels. Conversely, when NIZ were stimulated, 2.7% of SOZ channels showed decreased N1 amplitude and 1.5% showed increased N1 amplitude. All changes are reported in Figure 1 in detail. Overall, changes in N1 or N2 amplitudes changed more often than N1/N2 latency. Similarly, the N1 and N2 latency effects largely did not exceed what would be expected by chance in NIZ (NIZ to NIZ for N1 latency: 2.9%; for N2 latency: 2.9%). The highest rates of significant amplitude change were observed for stimulation–recording pairs in which both electrodes could be classified within the seizure network (i.e., SOZ and EPZ). For N1 amplitude, SOZ→SOZ pairs showed 15.9% significant change, EPZ→EPZ 15.6%, and IZ→IZ 11.6%. A similar pattern was observed for N2 amplitude (SOZ→SOZ: 15.5%; EPZ→EPZ: 11.1%; IZ→IZ: 12.4%). By contrast, NIZ→NIZ pairs showed significance rates of 5.7% and 7.4% for N1 and N2 amplitude respectively, approaching but modestly exceeding the FDR threshold, suggesting a limited but potentially real diffuse effect of ASMs on healthy tissue connectivity. However, when the direction of change was compared, the N1 amplitude was mostly decreased from ASM‐ON to ASM‐OFF in SOZ→SOZ and EPZ→EPZ compared to mostly an increase in N1 amplitude for the IZ→IZ pair.

N2 amplitude effects following SOZ stimulation demonstrated a clear directionality that varied systematically with the class of the recording electrode. When recording from EPZ, SOZ stimulation produced significant N2 amplitude increases in the ASM‐OFF condition in 9.2% of pairs, with only 2.5% showing decreases—the most unidirectional finding in the dataset. A similar but attenuated pattern was observed for SOZ→IZ pairs (5.3% increase vs. 2.2% decrease). Notably, the reverse was observed for SOZ→NIZ pairs, where N2 amplitude was more frequently larger in the ASM‐ON condition (4.7% decrease from ASM‐ON to ASM‐OFF) than in the ASM‐OFF condition (1.9% increase). A notable asymmetry was observed between SOZ→EPZ and EPZ→SOZ pairs. SOZ stimulation elicited significant N2 amplitude changes in EPZ recording electrodes (11.7% total significant, predominantly increases ASM‐OFF), whereas EPZ stimulation produced no significant N1 amplitude changes in SOZ recording electrodes (0.0%) and no significant N2 latency changes (0.0%).

**FIGURE 2:**
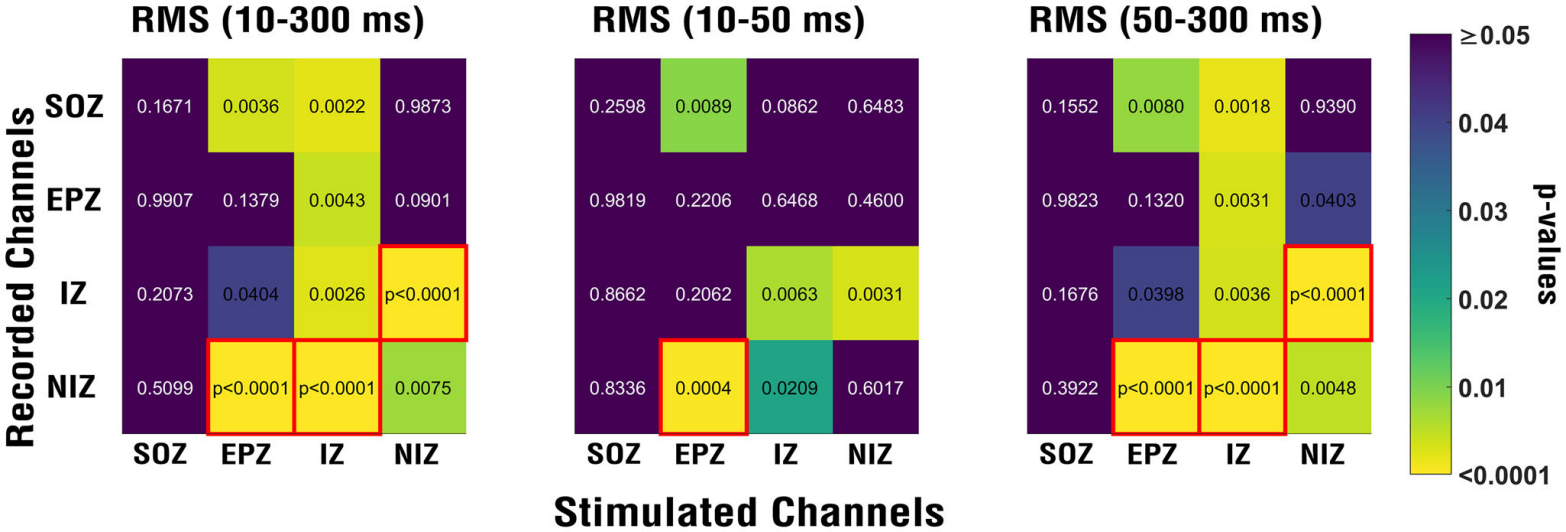
Changes in RMS values from ASM‐ON to ASM‐OFF. Each subplot contains results from a portion of the CCEP (10‐300 ms, 10–50 ms, and 50–300 ms). Stimulated channels are in columns and recording channels are in rows. Each box contains *p*‐values obtained from linear regression comparing the RMS values of each electrode pair across patients with ASM state and patient ID as random variables and the red frames indicate the significantly increased effective connectivity from ASM‐ON to ASM‐OFF state.

Since the RMS had to be calculated after obtaining the average CCEP waveform which resulted in only a single value per channel per condition, we statistically compared the change in RMS using linear regression with ASM‐state and patient ID as random variables (Figure 2). We conducted this analysis on RMS between 10 and 300 ms in addition to dividing this into two parts that correspond to the early and late CCEP response (10–50 ms and 50–300 ms, respectively). RMS at 10–300 ms period and RMS at 50–300 ms period were increased in NIZ>IZ, IZ>NIZ, and EPZ>NIZ directions (*p* < 0.0001). RMS at 10–50 ms period was increased from EPZ to NIZ (*p* = 0.0004).

### 3.3 Effects of ASMs on Functional Connectivity

**FIGURE 3:**
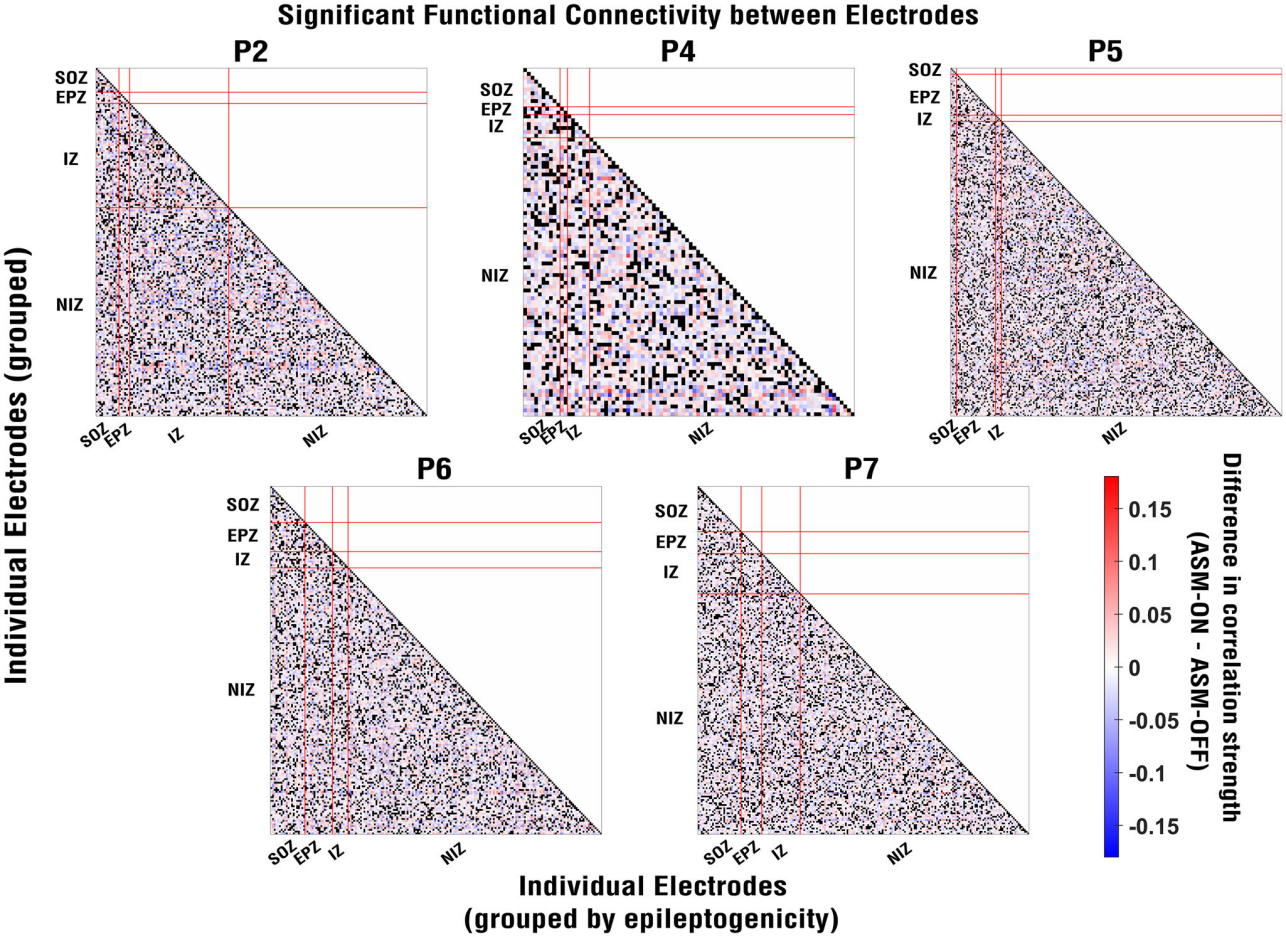
Changes in functional connectivity at individual electrode level. For five patients, we used rest recordings to calculate the functional connectivity from slow fluctuations of BHA. Each matrix shows the difference in connectivity strength between two sessions (subtracting ASM‐OFF from ASM‐ON) with rows and columns grouped by epileptogenicity as seizure onset zone (SOZ), epilepsy propagation zone (EPZ), irritative zone (IZ), and non‐involved zone (NIZ).Thus, the higher (red) values show higher connectivity during ASM‐ON than during ASM‐OFF session. Nonsignificant changes are masked with black.

**FIGURE 4:**
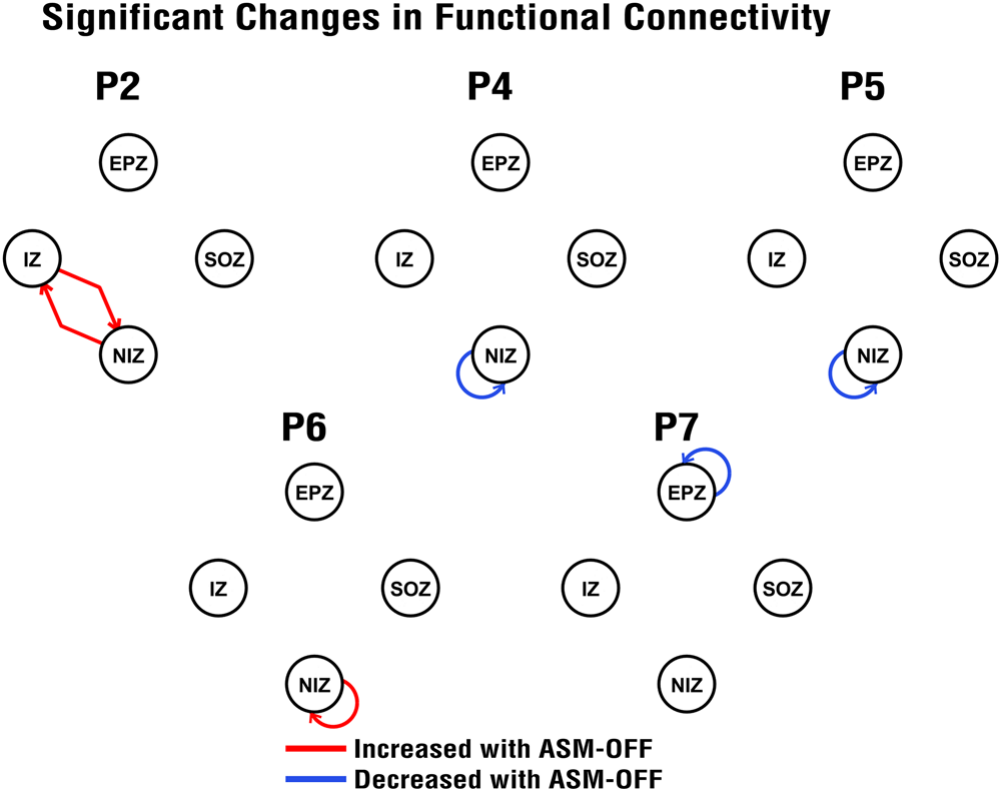
Significant changes in average functional connectivity based on epileptogenic profile. We calculated the functional connectivity between each pair of electrodes in five patients (P2, P4, P5, P6, and P7) and compared the average connectivity strength between two sessions using nonparametric permutation testing. Results of the significant connectivity changes from ASM‐ON to ASM‐OFF session are shown for each patient separately (*p < 0.05; **p < 0.01; ***p < 0.001).

We aimed to understand how ASMs affect functional brain networks. To this end, we calculated functional connectivity and several graph theoretical measures (shown in the next section) on five of the seven patients (P2, P4, P5, P6, and P7) both on and off ASMs in order to identify the changes in functional connectivity due to ASMs. Three patients had 1‐h resting state iEEG recordings prior to the SPES sessions and two had resting state recording at a random time with no seizures (other patients did not have this recording session). We calculated the slow fluctuations (0.1–1 Hz) of the BHA that is a reliable estimate of neuronal population spiking activity (Manning et al. 2009; Leszczyński et al. 2020). We then correlated the time series between each pair of electrodes to obtain functional connectivity. Permutation testing was used to calculate significant correlation at individual electrode level (Figure 3) and paired *t*‐tests were used to find the significant changes in group level (Figure 4).

At individual electrode level, we found significant changes in connectivity for most electrode pairs. The direction of the changes was not consistent across electrodes with connectivity decreasing in 39% of pairs and increasing in 42% of pairs from ASM‐ON to ASM‐OFF for P2, with 40% decreased and 39% increased for P4, 40% decreased and 38% increased for P5, 38% decreased and 41% increased for P6, and 39% decreased and 39% increased for P7 (Figure 3). Functional connectivity was increased between IZ and NIZ in either direction for P2 and within NIZ in P6 (*p* < 0.001) (Figure 4). In P4, P5, and P7, the functional connectivity was decreased within NIZ, NIZ, and EPZ, respectively (*p* < 0.001) (Figure 4).

### 3.4 Effects of ASMs on Graph Theoretical Measures

**FIGURE 5:**
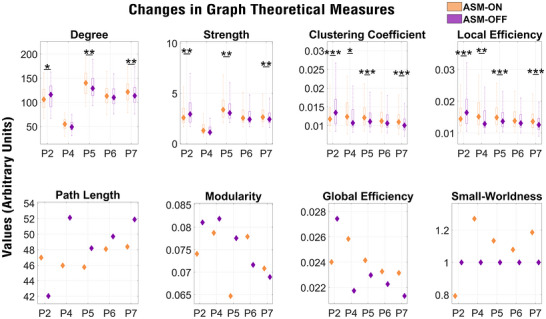
Changes in graph theoretical measures between ASM‐ON and ASM‐OFF sessions. We calculated the degree, strength, clustering coefficient, local efficiency, path length, modularity, global efficiency, and small‐worldness using the adjacency matrices derived from BHA‐based functional connectivity in five patients (P2, P4, P5, P6, and P7). We statistically compared the first four measures using Wilcoxon's sign‐rank test (*p < 0.05; **p < 0.01; ***p < 0.001). Due to the limited number of participants, we did not employ statistical analyses on path length, modularity, global efficiency, and small‐worldedness; however, it is depicted qualitatively.

We aimed at understanding the changes in network topology based on different brain states (ASM‐ON vs. ASM‐OFF). To this end, we calculated several graph theoretical measures from the functional connectivity adjacency matrices to compare how they change between different states (Figure 5). We found that the degree, strength, clustering coefficient, and local efficiency were significantly increased in P2 and decreased in P4 and P7 (*p* < 0.01 or 0 < 0.001). Additionally, clustering coefficient and local efficiency were decreased in P4 (*p* < 0.05 or 0 < 0.01).

## 4 Discussion

While ASMs ideally influence epileptic tissue more than non‐epileptic brain areas, there is scant evidence on how ASMs alter brain activity in the human brain. We used a unique opportunity to assess the effect of ASMs on both functional and effective brain networks during iEEG monitoring with seven patients. First, we found that ASMs altered the effective connectivity within the seizure network (SOZ and EPZ) more often than with NIZ or IZ. In five of these patients, we analyzed functional connectivity and graph theoretical measures from 1‐h rest recordings showing diverse changes between two sessions.

ASMs have multiple mechanisms of action which result in different propagation patterns and strength of epileptic and non‐epileptic signals within the brain (Khateb et al. 2021). For example, a study on healthy volunteers showed that carbamazepine and OXC resulted in slowing of visual evoked potential; however, LEV did not cause any neurophysiological change (Mecarelli et al. 2004). While most iEEG studies do not control for ASMs (Groppe et al. 2013), several studies found that the power in delta, theta, and alpha frequency bands was decreased along with decrease in spike frequencies with ASM taper off (Spencer et al. 2008; Zaveri et al. 2010; Goncharova et al. 2013; Goncharova et al. 2016; Meisel et al. 2015). Similar findings were also shown in chronic electrode implantation with responsive neurostimulation where the spike frequencies and wideband power have decreased after initiation of ASMs with multiple mechanisms (Skarpaas et al. 2018). While our functional connectivity measure was based on the BHA, which may reflect the spike frequencies, we did not observe a predilection of the BHA‐based functional connectivity to increase during ASM‐OFF session (Figure 3).

CCEPs have been used to describe the effective connectivity between functionally connected brain regions (Matsumoto et al. 2004, 2007). It has been shown that the location and amplitude of the CCEPs were affected by whether the recording or the stimulated electrodes were in the SOZ (Valentin 2002; Enatsu et al. 2012). It has also been shown that the CCEPs were affected by the brain state including consciousness and anesthesia (Usami et al. 2015; Suzuki et al. 2019; Zelmann et al. 2023). However, the effect of ASMs on the CCEPs has not been previously shown. We found that the amplitude and latencies of the N1 and N2 responses were altered more within the seizure network (SOZ and EPZ) than the NIZ and IZ. This is in line with previous work showing decrease in spike frequency with ASM taper (Spencer et al. 2008; Zaveri et al. 2010; Goncharova et al. 2013; Goncharova et al. 2016). The relative sparing of NIZ pairs (significance rates approached but only modestly exceeded FDR threshold) suggests that ASM effects on NIZ effective connectivity are limited. This has potential clinical relevance: It implies that the therapeutic window of ASMs may partly reflect their selectivity for pathological over physiological network dynamics, and that CCEPs could serve as a sensitive, spatially resolved marker of this selectivity in individual patients. Additionally, it was previously shown that the RMS‐based mapping of seizure network reflected early versus late spread pattern of seizures (Lega et al. 2015). Here, we found that the alterations in RMS are limited to EPZ/IZ→NIZ or NIZ→IZ (Figure 2). We also acknowledge that the number of stimulated electrodes were chosen by the clinical team, thus NIZ and IZ were not as clinically relevant for stimulation. This imbalance across patients may have led to difference in results between effective and functional connectivity.

The dissociation in the direction of amplitude change between SOZ/EPZ and IZ is among the most important findings of this study. Within the SOZ and EPZ, ASM withdrawal was associated with a net reduction in N1 amplitude (i.e., the responses were paradoxically smaller off medication than on) suggesting that ASMs increase local excitability within these regions rather than suppress it in a straightforward manner. This seemingly counterintuitive finding may reflect the complex excitation/inhibition balance such that excitatory and inhibitory populations are tightly coupled where a pharmacological enhancement of inhibition paradoxically increases the gain of the network's response to external input by stabilizing otherwise chaotic or suppressed excitatory dynamics (Sadeh and Clopath 2021). It has been proposed that in chronically epileptic networks, GABAergic interneurons are themselves dysfunctional or depleted, such that drugs enhancing inhibition (e.g., sodium channel blockers reducing pyramidal cell firing) may paradoxically increase the net evoked response amplitude by reducing surround inhibition or unmasking excitatory recurrent connections (de Curtis and Avanzini 2001). In contrast, the IZ showed the opposite pattern (i.e., larger amplitudes off of medication) which may reflect a different cellular substrate in peri‐ictal tissue that retains more intact inhibitory machinery and responds to ASM withdrawal with net disinhibition rather than reduced excitability. Disentangling these mechanisms would require concurrent local field potential recordings or laminar analysis beyond the scope of the current dataset, but the directional dissociation itself provides a clear empirical basis for distinguishing SOZ/EPZ from IZ at the network level. The finding regarding the asymmetry between SOZ and EPZ where SOZ stimulation produced consistent N2 amplitude changes in EPZ electrodes, but not the opposite lends electrophysiological support to the concept of the SOZ as a network hub with preferential efferent connectivity into surrounding epileptic tissue. This hierarchical organization is consistent with models of seizure initiation in which a discrete generating zone recruits adjacent irritative tissue through feedforward excitation rather than through reciprocal or feedback‐dominated dynamics (Bartolomei et al. 2017; Proix et al. 2018).

Effective connectivity is a measure of causal influence of one brain region toward the other. CCEPs closely reflect the structural and directed connectivity between stimulated and recorded brain regions (Conner et al. 2011; Crocker et al. 2021). On the other hand, functional connectivity can be studied with task or rest recordings and is mainly related to the statistical, undirected interdependencies between brain regions and does not necessarily rely on the structural connectivity (Biswal et al. 1995). When analyzed within participants, it has previously been shown that, although significantly correlated, the N2 of CCEPs and fMRI‐based functional connectivity measures do not fully overlap (Keller et al. 2011). Similarly, iEEG‐based functional connectivity is known to be correlated with CCEP and the correlation depends on the distance between regions (Keller et al. 2014a). These differences between effective and functional connectivity could partly explain the differences of the effects of ASMs. Overall, CCEPs reflect causal influence of brain regions onto each other, thus more closely related to spread of epileptic activity. On the other hand, functional connectivity measures do not necessarily rely on direct connection between brain regions, thus opening the door for potential influence of a third region onto the two brain regions analyzed, where the third region might be the epileptic region. It can be argued that removing the effect of epileptic activity on healthy brain regions with ASMs may result in changes in functional connectivity measures (Paulo et al. 2022).

Multiple studies using the interictal iEEG‐based functional connectivity or graph theoretical measures found that mesial temporal lobe structures in mesial temporal lobe epilepsy had increased synchrony within these structures (Schevon et al. 2007; Bettus et al. 2008; Bartolomei et al. 2013). Recently, there have been several studies analyzing the changes in functional connectivity due to ASMs. Paulo et al. (2022) calculated different coherence measures using 2 min iEEG epochs while patients were weaned off of ASMs after surgical implantation of intracranial electrodes. They found that the inward and nondirected functional connectivity was different between the epileptogenic zone and non‐epileptogenic regions, and more importantly, the difference in between‐imaginary coherence between epileptogenic and non‐epileptogenic regions was not as pronounced with lower doses of ASMs. In our study, we found that the functional connectivity within non‐epileptogenic brain areas was altered off ASMs, but not within epileptogenic brain regions, while there was no consistent effect given our limitation with five patients.

Graph theoretical measures can explain part of the difference between how ASMs show different cognitive side effects. Most graph theoretical measures used fMRI which is limited in temporal and spatial resolution compared to iEEG, and iEEG‐based graph theoretical measures have not been used previously to show changes in these measures based on ASM status (Rubinov and Sporns 2010; Panzica et al. 2013; Bartolomei et al. 2013). In addition to the SPES session, we were able to record resting iEEG in five patients. Overall, these cases had opposite results in graph theoretical measures, specifically in degree, strength, clustering coefficient, and local efficiency. One possible reason is that P4 did not have any habitual seizures while being off ASMs, which might partly explain the network differences. It is also known that during ASM taper, spike rate decreases which may drive part of the connectivity results (Spencer et al. 2008; Goncharova et al. 2016). Another potential explanation is that the locations of SOZ were different across patients, which have different connectivity patterns and might be affected differently by the ASMs (Bettus et al. 2008). A recent prospective study identified the effects of ASMs on functional MRI‐based functional connectivity profiles after first time seizure and starting an ASM, and found alterations in graph theoretical measures of network connectivity more prominently in patients with uncontrolled seizures (Pedersen et al. 2024). However, their findings may have been driven by the seizure recurrence, but not the ASMs themselves.

Our study is limited by several factors, mainly the number of participants and channels stimulated during both ASM‐ON and ASM‐OFF sessions. Unfortunately, we were not able to evaluate the possible use of CCEPs to localize the epileptogenic network due to the limited number of stimulation sessions that were done as part of the clinical care. Additionally, we did not have enough power to predict if the patient is on ASM or not solely based on iEEG features. In future studies, N1 and N2 responses can be used to cluster the SOZ channels from NIZ channels. While SPES was used to lower the seizure threshold of the patients, a seizure was induced during SPES session in two patients (P3 and P7) and the stimulated pair‐inducing seizure was excluded from analysis. This also shows safety of SPES in seizure induction, but the knowledge in this area is limited (Trébuchon and Chauvel 2016; Cuello Oderiz et al. 2019; Arya et al. 2025). Another limitation of our study is that we did not control for the patient's state (i.e., sleep, awake), which may account for a part of the differences, as this was shown to affect CCEP parameters (Suzuki et al. 2019; Zelmann et al. 2023). Generalizability of our study is inherently limited to medically refractory epilepsy patients since CCEPs can only be recorded in this population and functional connectivity may differ in medically refractory epilepsy patients (Pressl et al. 2019). We also acknowledge that the variety of the ASMs that the patients were on prevented us from weighing or controlling the effective or functional connectivity measures based on differences in ASMs.

## 5 Conclusion

In summary, we found that the ASMs altered effective connectivity more within the seizure network, but the alterations in functional connectivity were outside the seizure network. Given this is a unique opportunity to study effects of ASMs, we were limited by the number of patients we analyzed. These findings support the use of CCEPs not only as a localization tool, but as a pharmacodynamic readout capable of characterizing the network‐level effects of ASMs with spatial and directional resolution that is not accessible through scalp EEG or neuroimaging alone. Future studies are needed to expand the changes in CCEPs and functional connectivity to tie the alterations to specific medication or mechanism of actions, to uncover if certain functional networks are more vulnerable to certain medications, and to find if different iEEG measures including CCEPs and functional connectivity methods can be used to guide medication selection or to localize seizure network based on changes in ASMs.

## Author Contributions

**Helen E. Brinyark**: writing – review and editing, conceptualization, data curation, project administration. **Rebekah Chatfield**: data curation, methodology, project administration, writing – review and editing. **Serdar Akkol**: conceptualization, methodology, formal analysis, visualization, writing – review and editing, writing – original draft, investigation. **Rachel June Smith**: funding acquisition, writing – review and editing, methodology, conceptualization, data curation, supervision, investigation. **Aparna Vaddiparti**: writing – review and editing, methodology, project administration. **Benjamin C. Cox**: supervision, writing – review and editing, conceptualization, methodology, funding acquisition, investigation.

## Funding

This work is supported by the American Epilepsy Society Junior Investigator Award (1042632) and the CURE Epilepsy Taking Flight Award (1061181), both to R.J.S.

## Conflicts of Interest

The authors declare no conflicts of interest.

## Data Availability

The code used for analyses will be available at: https://github.com/sakkol/CCEP_ASM_connectivity. The dataset used for the current study is available from the corresponding author on reasonable request. The data are not publicly available due to privacy or ethical restrictions.

## References

[brb371507-bib-0001] Akkol, S. , A. Kucyi , W. Hu , et al. 2021. “Intracranial Electroencephalography Reveals Selective Responses to Cognitive Stimuli in the Periventricular Heterotopias.” Journal of Neuroscience 41: 3870–3878. 10.1523/JNEUROSCI.2785-20.2021. 33727335 PMC8084321

[brb371507-bib-0002] Arya, R. , F. M. Baumer , P. Chauvel , et al. 2025. “American Clinical Neurophysiology Society Technical Standards for Electrical Stimulation With Intracranial Electrodes for Functional Brain Mapping and Seizure Induction.” Journal of Clinical Neurophysiology 42: 190–200. 10.1097/WNP.0000000000001149. 39946166

[brb371507-bib-0003] Bartolomei, F. , G. Bettus , C. J. Stam , and M. Guye . 2013. “Interictal Network Properties in Mesial Temporal Lobe Epilepsy: A Graph Theoretical Study From Intracerebral Recordings.” Clinical Neurophysiology 124: 2345–2353. 10.1016/j.clinph.2013.06.003. 23810635

[brb371507-bib-0004] Bauer, A. Q. , A. W. Kraft , G. A. Baxter , et al. 2018. “Effective Connectivity Measured Using Optogenetically Evoked Hemodynamic Signals Exhibits Topography Distinct From Resting State Functional Connectivity in the Mouse.” Cerebral Cortex 28: 370–386. 10.1093/cercor/bhx298. 29136125 PMC6057523

[brb371507-bib-0005] Beghi, E. , G. Giussani , E. Nichols , et al. 2019. “Global, Regional, and National Burden of Epilepsy, 1990–2016: A Systematic Analysis for the Global Burden of Disease Study 2016.” Lancet Neurology 18: 357–375. 10.1016/S1474-4422(18)30454-X. 30773428 PMC6416168

[brb371507-bib-0006] Bettus, G. , F. Wendling , M. Guye , et al. 2008. “Enhanced EEG Functional Connectivity in Mesial Temporal Lobe Epilepsy.” Epilepsy Research 81: 58–68. 10.1016/j.eplepsyres.2008.04.020. 18547787

[brb371507-bib-0007] Biswal, B. , F. Zerrin Yetkin , V. M. Haughton , and J. S. Hyde . 1995. “Functional Connectivity in the Motor Cortex of Resting Human Brain Using Echo‐Planar MRI.” Magnetic Resonance in Medicine 34: 537–541. 10.1002/mrm.1910340409. 8524021

[brb371507-bib-0008] Boccaletti, S. , V. Latora , Y. Moreno , M. Chavez , D. Hwang , et al. 2006. “Complex Networks: Structure and Dynamics.” Physics Reports 424: 175–308. 10.1016/j.physrep.2005.10.009.

[brb371507-bib-0069] Bartolomei, F. , S. Lagarde , F. Wendling , et al. 2017. “Defining epileptogenic networks: contribution of SEEG and signal analysis.” Epilepsia 58: 1131–1147. 10.1111/epi.1379. 28543030

[brb371507-bib-0009] Canevini, M. P. , G. De Sarro , C. A. Galimberti , et al. 2010. “Relationship Between Adverse Effects of Antiepileptic Drugs, Number of Coprescribed Drugs, and Drug Load in a Large Cohort of Consecutive Patients With Drug‐Refractory Epilepsy.” Epilepsia 51: 797–804. 10.1111/j.1528-1167.2010.02520.x. 20545754

[brb371507-bib-0010] Cao, J. , Y. Zhao , X. Shan , et al. 2022. “Brain Functional and Effective Connectivity Based on Electroencephalography Recordings: A Review.” Human Brain Mapping 43: 860–879. 10.1002/hbm.25683. 34668603 PMC8720201

[brb371507-bib-0011] Chen, Z. , M. J. Brodie , D. Liew , and P. Kwan . 2018. “Treatment Outcomes in Patients With Newly Diagnosed Epilepsy Treated With Established and New Antiepileptic Drugs: A 30‐Year Longitudinal Cohort Study.” JAMA Neurology 75: 279. 10.1001/jamaneurol.2017.3949. 29279892 PMC5885858

[brb371507-bib-0012] Conner, C. R. , T. M. Ellmore , M. A. DiSano , et al. 2011. “Anatomic and Electro‐Physiologic Connectivity of the Language System: A Combined DTI‐CCEP Study.” Computers in Biology and Medicine 41: 1100–1109. 10.1016/j.compbiomed.2011.07.008. 21851933 PMC3223284

[brb371507-bib-0013] Crocker, B. , L. Ostrowski , Z. M. Williams , et al. 2021. “Local and Distant Responses to Single Pulse Electrical Stimulation Reflect Different Forms of Connectivity.” Neuroimage 237: 118094. 10.1016/j.neuroimage.2021.118094. 33940142 PMC12424142

[brb371507-bib-0014] Cuello Oderiz, C. , N. von Ellenrieder , F. Dubeau , et al. 2019. “Association of Cortical Stimulation–Induced Seizure With Surgical Outcome in Patients With Focal Drug‐Resistant Epilepsy.” JAMA Neurology 76: 1070. 10.1001/jamaneurol.2019.1464. 31180505 PMC6563597

[brb371507-bib-0015] Czuczwar, S. J. , J. Kaplanski , G. Swiderska‐Dziewit , et al. 2009. “Pharmacodynamic Interactions Between Antiepileptic Drugs: Preclinical Data Based on Isobolography.” Expert Opinion on Drug Metabolism & Toxicology 5: 131–136. 10.1517/17425250802677826. 19210232

[brb371507-bib-0016] de Curtis, M. , and G. Avanzini . 2001. “Interictal Spikes in Focal Epileptogenesis.” Progress in Neurobiology 63: 541–567. 10.1016/S0301-0082(00)00026-5. 11164621

[brb371507-bib-0017] Enatsu, R. , K. Jin , S. Elwan , et al. 2012. “Correlations Between Ictal Propagation and Response to Electrical Cortical Stimulation: A Cortico‐Cortical Evoked Potential Study.” Epilepsy Research 101: 76–87. 10.1016/j.eplepsyres.2012.03.004. 22459638

[brb371507-bib-0018] Enatsu, R. , Y. Kubota , Y. Kakisaka , et al. 2013. “Reorganization of Posterior Language Area in Temporal Lobe Epilepsy: A Cortico‐Cortical Evoked Potential Study.” Epilepsy Research 103: 73–82. 10.1016/j.eplepsyres.2012.07.008. 22819071

[brb371507-bib-0019] Feigin, V. L. , A. A. Abajobir , K. H. Abate , et al. 2017. “Global, Regional, and National Burden of Neurological Disorders During 1990–2015: A Systematic Analysis for the Global Burden of Disease Study 2015.” Lancet Neurology 16: 877–897. 10.1016/S1474-4422(17)30299-5. 28931491 PMC5641502

[brb371507-bib-0020] Friston, K. J. 2011. “Functional and Effective Connectivity: A Review.” Brain Connectivity 1: 13–36. 10.1089/brain.2011.0008. 22432952

[brb371507-bib-0021] Goncharova, I. I. , R. Alkawadri , N. Gaspard , et al. 2016. “The Relationship Between Seizures, Interictal Spikes and Antiepileptic Drugs.” Clinical Neurophysiology 127: 3180–3186. 10.1016/j.clinph.2016.05.014. 27292227

[brb371507-bib-0022] Goncharova, I. I. , S. S. Spencer , R. B. Duckrow , et al. 2013. “Intracranially Recorded Interictal Spikes: Relation to Seizure Onset Area and Effect of Medication and Time of Day.” Clinical Neurophysiology 124: 2119–2128. 10.1016/j.clinph.2013.05.027. 23856192

[brb371507-bib-0023] Groppe, D. M. , S. Bickel , C. J. Keller , et al. 2013. “Dominant Frequencies of Resting Human Brain Activity as Measured by the Electrocorticogram.” Neuroimage 79: 223–233. 10.1016/j.neuroimage.2013.04.044. 23639261 PMC4269223

[brb371507-bib-0024] Haneef, Z. , H. S. Levin , and S. Chiang . 2015. “Brain Graph Topology Changes Associated With Anti‐Epileptic Drug Use.” Brain Connectivity 5: 284–291. 10.1089/brain.2014.0304. 25492633 PMC4490704

[brb371507-bib-0025] Ilmoniemi, R. J. , J. Virtanen , J. Ruohonen , et al. 1997. “Neuronal Responses to Magnetic Stimulation Reveal Cortical Reactivity and Connectivity.” Neuroreport 8: 3537–3540. 9427322 10.1097/00001756-199711100-00024

[brb371507-bib-0026] Jokeit, H. , M. Okujava , and F. G. Woermann . 2001. “Carbamazepine Reduces Memory Induced Activation of Mesial Temporal Lobe Structures: A Pharmacological fMRI‐Study.” BMC Neurology 1: 6. 10.1186/1471-2377-1-6. 11710962 PMC59836

[brb371507-bib-0027] Keller, C. J. , S. Bickel , L. Entz , et al. 2011. “Intrinsic Functional Architecture Predicts Electrically Evoked Responses in the Human Brain.” Proceedings of the National Academy of Sciences 108: 10308–10313. 10.1073/pnas.1019750108. PMC3121855 21636787

[brb371507-bib-0028] Keller, C. J. , S. Bickel , C. J. Honey , et al. 2013. “Neurophysiological Investigation of Spontaneous Correlated and Anticorrelated Fluctuations of the BOLD Signal.” Journal of Neuroscience 33: 6333–6342. 10.1523/JNEUROSCI.4837-12.2013. 23575832 PMC3652257

[brb371507-bib-0029] Keller, C. J. , C. J. Honey , L. Entz , et al. 2014a. “Corticocortical Evoked Potentials Reveal Projectors and Integrators in Human Brain Networks.” Journal of Neuroscience 34: 9152–9163. 10.1523/JNEUROSCI.4289-13.2014. 24990935 PMC4078089

[brb371507-bib-0030] Keller, C. J. , C. J. Honey , P. Mégevand , et al. 2014b. “Mapping Human Brain Networks With Cortico‐Cortical Evoked Potentials.” Philosophical Transactions of the Royal Society B: Biological Sciences 369: 20130528. 10.1098/rstb.2013.0528. PMC4150303 25180306

[brb371507-bib-0031] Khateb, M. , N. Bosak , and M. Herskovitz . 2021. “The Effect of Anti‐Seizure Medications on the Propagation of Epileptic Activity: A Review.” Frontiers in Neurology 12: 674182. 10.3389/fneur.2021.674182. 34122318 PMC8191738

[brb371507-bib-0032] Kucyi, A. , J. Schrouff , S. Bickel , et al. 2018. “Intracranial Electrophysiology Reveals Reproducible Intrinsic Functional Connectivity Within Human Brain Networks.” Journal of Neuroscience 38: 4230–4242. 10.1523/JNEUROSCI.0217-18.2018. 29626167 PMC5963853

[brb371507-bib-0033] Lega, B. , S. Dionisio , P. Flanigan , et al. 2015. “Cortico‐Cortical Evoked Potentials for Sites of Early Versus Late Seizure Spread in Stereoelectroencephalography.” Epilepsy Research 115: 17–29. 10.1016/j.eplepsyres.2015.04.009. 26220373

[brb371507-bib-0034] Leszczyński, M. , A. Barczak , Y. Kajikawa , et al. 2020. “Dissociation of Broadband High‐Frequency Activity and Neuronal Firing in the Neocortex.” Science Advances 6: eabb0977. 32851172 10.1126/sciadv.abb0977 PMC7423365

[brb371507-bib-0035] Manning, J. R. , J. Jacobs , I. Fried , and M. J. Kahana . 2009. “Broadband Shifts in Local Field Potential Power Spectra Are Correlated With Single‐Neuron Spiking in Humans.” Journal of Neuroscience 29: 13613–13620. 10.1523/JNEUROSCI.2041-09.2009. 19864573 PMC3001247

[brb371507-bib-0036] Matsumoto, R. , T. Kunieda , and D. Nair . 2017. “Single Pulse Electrical Stimulation to Probe Functional and Pathological Connectivity in Epilepsy.” Seizure 44: 27–36. 10.1016/j.seizure.2016.11.003. 27939100 PMC5291825

[brb371507-bib-0037] Matsumoto, R. , D. R. Nair , E. LaPresto , et al. 2004. “Functional Connectivity in the Human Language System: A Cortico‐Cortical Evoked Potential Study.” Brain 127: 2316–2330. 10.1093/brain/awh246. 15269116

[brb371507-bib-0038] Matsumoto, R. , D. R. Nair , E. LaPresto , et al. 2007. “Functional Connectivity in Human Cortical Motor System: A Cortico‐Cortical Evoked Potential Study.” Brain 130: 181–197. 10.1093/brain/awl257. 17046857

[brb371507-bib-0039] Mecarelli, O. , E. Vicenzini , P. Pulitano , et al. 2004. “Clinical, Cognitive, and Neurophysiologic Correlates of Short‐Term Treatment With Carbamazepine, Oxcarbazepine, and Levetiracetam in Healthy Volunteers.” Annals of Pharmacotherapy 38: 1816–1822. 10.1345/aph.1E136. 15367726

[brb371507-bib-0040] Meisel, C. , A. Schulze‐Bonhage , D. Freestone , et al. 2015. “Intrinsic Excitability Measures Track Antiepileptic Drug Action and Uncover Increasing/Decreasing Excitability Over the Wake/Sleep Cycle.” Proceedings of the National Academy of Sciences 112: 14694–14699. 10.1073/pnas.1513716112. PMC4664307 26554021

[brb371507-bib-0041] Mohanty, R. , W. A. Sethares , V. A. Nair , and V. Prabhakaran . 2020. “Rethinking Measures of Functional Connectivity via Feature Extraction.” Scientific Reports 10: 1298. 10.1038/s41598-020-57915-w. 31992762 PMC6987226

[brb371507-bib-0042] Nir, Y. , R. Mukamel , I. Dinstein , et al. 2008. “Interhemispheric Correlations of Slow Spontaneous Neuronal Fluctuations Revealed in Human Sensory Cortex.” Nature Neuroscience 11: 1100–1108. 10.1038/nn.2177. 19160509 PMC2642673

[brb371507-bib-0043] Oostenveld, R. , P. Fries , E. Maris , and J.‐M. Schoffelen . 2011. “FieldTrip: Open Source Software for Advanced Analysis of MEG, EEG, and Invasive Electrophysiological Data.” Computational Intelligence and Neuroscience 2011: 156869. 10.1155/2011/156869. 21253357 PMC3021840

[brb371507-bib-0044] Panzica, F. , G. Varotto , F. Rotondi , R. Spreafico , S. Franceschetti , et al. 2013. “Identification of the Epileptogenic Zone From Stereo‐EEG Signals: A Connectivity‐Graph Theory Approach.” Frontiers in Neurology 4: 175. 10.3389/fneur.2013.00175. 24223569 PMC3818576

[brb371507-bib-0045] Paulo, D. L. , K. E. Wills , G. W. Johnson , et al. 2022. “SEEG Functional Connectivity Measures to Identify Epileptogenic Zones.” Neurology 98: e2060–e2072. 10.1212/WNL.0000000000200386. 35338075 PMC9162047

[brb371507-bib-0046] Pedersen, M. , H. Pardoe , R. Mito , et al. 2024. “Brain Network Changes After the First Seizure: An Insight Into Medication Response?.” Brain Communications 6: fcae328. 10.1093/braincomms/fcae328. 39440302 PMC11495098

[brb371507-bib-0047] Perucca, E. , and P. Kwan . 2005. “Overtreatment in Epilepsy.” CNS Drugs 19: 897–908. 10.2165/00023210-200519110-00001. 16268662

[brb371507-bib-0048] Perucca, P. , and F. G. Gilliam . 2012. “Adverse Effects of Antiepileptic Drugs.” Lancet Neurology 11: 792–802. 10.1016/S1474-4422(12)70153-9. 22832500

[brb371507-bib-0049] Pressl, C. , P. Brandner , S. Schaffelhofer , et al. 2019. “Resting State Functional Connectivity Patterns Associated With Pharmacological Treatment Resistance in Temporal Lobe Epilepsy.” Epilepsy Research 149: 37–43. 10.1016/j.eplepsyres.2018.11.002. 30472489 PMC6483378

[brb371507-bib-0050] Prime, D. , M. Woolfe , D. Rowlands , S. O'Keefe , S. Dionisio , et al. 2020. “Comparing Connectivity Metrics in Cortico‐Cortical Evoked Potentials Using Synthetic Cortical Response Patterns.” Journal of Neuroscience Methods 334: 108559. 10.1016/j.jneumeth.2019.108559. 31927000

[brb371507-bib-0070] Proix, T. , V. K. Jirsa , F. Bartolomei . et al. 2018. “Predicting the spatiotemporal diversity of seizure propagation and termination in human focal epilepsy.” Nat Commun 9: 1088. 10.1038/s41467-018-02973-y. 29540685 PMC5852068

[brb371507-bib-0051] Rubinov, M. , and O. Sporns . 2010. “Complex Network Measures of Brain Connectivity: Uses and Interpretations.” Neuroimage 52: 1059–1069. 10.1016/j.neuroimage.2009.10.003. 19819337

[brb371507-bib-0052] Sadeh, S. , and C. Clopath . 2021. “Inhibitory Stabilization and Cortical Computation.” Nature Reviews Neuroscience 22: 21–37. 10.1038/s41583-020-00390-z. 33177630

[brb371507-bib-0053] Sammarra, I. , M. E. Caligiuri , M. C. Bonacci , et al. 2024. “May Anti‐Seizure Medications Alter Brain Structure in Temporal Lobe Epilepsy? A Prospective Study.” Epilepsia Open 9: 1076–1082. 10.1002/epi4.12912. 38475905 PMC11145604

[brb371507-bib-0054] Schevon, C. A. , J. Cappell , R. Emerson , et al. 2007. “Cortical Abnormalities in Epilepsy Revealed by Local EEG Synchrony.” Neuroimage 35: 140–148. 10.1016/j.neuroimage.2006.11.009. 17224281 PMC1994936

[brb371507-bib-0055] Sills, G. J. , and M. A. Rogawski . 2020. “Mechanisms of Action of Currently Used Antiseizure Drugs.” Neuropharmacology 168: 107966. 10.1016/j.neuropharm.2020.107966. 32120063

[brb371507-bib-0056] Skarpaas, T. L. , T. K. Tcheng , and M. J. Morrell . 2018. “Clinical and Electrocorticographic Response to Antiepileptic Drugs in Patients Treated With Responsive Stimulation.” Epilepsy & Behavior 83: 192–200. 10.1016/j.yebeh.2018.04.003. 29719278

[brb371507-bib-0057] Spencer, S. S. , I. I. Goncharova , R. B. Duckrow , E. J. Novotny , H. P. Zaveri , et al. 2008. “Interictal Spikes on Intracranial Recording: Behavior, Physiology, and Implications.” Epilepsia 49: 1881–1892. 10.1111/j.1528-1167.2008.01641.x. 18479398

[brb371507-bib-0058] Stam, C. J. , and J. C. Reijneveld . 2007. “Graph Theoretical Analysis of Complex Networks in the Brain.” Nonlinear Biomedical Physics 1: 3. 10.1186/1753-4631-1-3. 17908336 PMC1976403

[brb371507-bib-0059] Suzuki, Y. , R. Enatsu , A. Kanno , et al. 2019. “The Influence of Anesthesia on Corticocortical Evoked Potential Monitoring Network Between Frontal and Temporoparietal Cortices.” World Neurosurgery 123: e685–e692. 10.1016/j.wneu.2018.11.253. 30576824

[brb371507-bib-0060] Szaflarski, J. P. , and J. B. Allendorfer . 2012. “Topiramate and Its Effect on fMRI of Language in Patients With Right or Left Temporal Lobe Epilepsy.” Epilepsy & Behavior 24: 74–80. 10.1016/j.yebeh.2012.02.022. 22481042 PMC3564045

[brb371507-bib-0061] Trébuchon, A. , and P. Chauvel . 2016. “Electrical Stimulation for Seizure Induction and Functional Mapping in Stereoelectroencephalography.” Journal of Clinical Neurophysiology 33: 511–521. 10.1097/WNP.0000000000000313. 27918346

[brb371507-bib-0062] Usami, K. , R. Matsumoto , K. Kobayashi , et al. 2015. “Sleep Modulates Cortical Connectivity and Excitability in Humans: Direct Evidence From Neural Activity Induced by Single‐Pulse Electrical Stimulation.” Human Brain Mapping 36: 4714–4729. 10.1002/hbm.22948. 26309062 PMC6869089

[brb371507-bib-0063] Valentin, A. 2002. “Responses to Single Pulse Electrical Stimulation Identify Epileptogenesis in the Human Brain In Vivo.” Brain 125: 1709–1718. 10.1093/brain/awf187. 12135963

[brb371507-bib-0064] Wandschneider, B. , J. Burdett , L. Townsend , et al. 2017. “Effect of Topiramate and Zonisamide on fMRI Cognitive Networks.” Neurology 88: 1165–1171. 10.1212/WNL.0000000000003736. 28213372 PMC5373787

[brb371507-bib-0065] Wandschneider, B. , and M. J. Koepp . 2016. “Pharmaco fMRI: Determining the Functional Anatomy of the Effects of Medication.” NeuroImage: Clinical 12: 691–697. 10.1016/j.nicl.2016.10.002. 27766202 PMC5067101

[brb371507-bib-0066] Wandschneider, B. , J. Stretton , M. Sidhu , et al. 2014. “Levetiracetam Reduces Abnormal Network Activations in Temporal Lobe Epilepsy.” Neurology 83: 1508–1512. 10.1212/WNL.0000000000000910. 25253743 PMC4222853

[brb371507-bib-0067] Zaveri, H. P. , S. M. Pincus , I. I. Goncharova , et al. 2010. “Background Intracranial EEG Spectral Changes With Anti‐Epileptic Drug Taper.” Clinical Neurophysiology 121: 311–317. 10.1016/j.clinph.2009.11.081. 20075002

[brb371507-bib-0068] Zelmann, R. , A. C. Paulk , F. Tian , et al. 2023. “Differential Cortical Network Engagement During States of Un/Consciousness in Humans.” Neuron 111: 3479–3495.e6. 10.1016/j.neuron.2023.08.007. 37659409 PMC10843836

